# CT perfusion identified potential treatment opportunities in one in five mild strokes

**DOI:** 10.1186/s42466-025-00442-8

**Published:** 2025-11-07

**Authors:** Yohanna Kusuma, Bizhong Che, Presaad Pillai, Ximing Nie, Leonard Yeo LL, Vijay K. Sharma, Andrew Wong, Peter Riley, Benjamin Clissold, Paul Talman, Mursyid Bustami, Lyna Soertidewi, M. Arief R. Kemal, Indah A. Putri, Reza Aditya Arpandy, Nandini Phalita Laksmi, Nurul Rakhmawati, Paul Yielder, Bernard Yan

**Affiliations:** 1 Present Address: School of Medicine, Deakin University, Waurn Ponds, VIC Australia; 2 The First Affiliated Hospital of Xi’an Jiaotong University, International Obesity and Metabolic Disease Research Centre, Xi’an, Shaanxi P. R. China; 3 Present Address: Department of Neurology, The Royal Melbourne Hospital, Melbourne, VIC Australia; 4 Present Address: Faculty of Medicine, Dentistry and Health Sciences, The University of Melbourne, Melbourne, Australia; 5 Department of Neurology, The Geelong University Hospital, Geelong, VIC Australia; 6 Department of Neurology, Royal Brisbane and Women’s Hospital, Brisbane, QLD Australia; 7 Yong Loo Lin School of Medicine, National University of Singapore, Singapore, Singapore; 8 Division of Neurology, National University Health System, Singapore, Singapore; 9 Present Address: Department of Neurology, National Brain Centre Prof Dr. dr Mahar Mardjono-Airlangga University, Jakarta/Surabaya, Indonesia; 10 Ontario Tech University Oshawa, Oshawa, ON Canada; 11 Queen Elizabeth Hospital II, Sabah, Malaysia; 12 Dept. Neurology, Beijing Tiantan Hospital, Capital Medical University, Beijing, China; 13 Neurology, Melbourne Brain Centre - The Royal Melbourne Hospital School of Medicine Deakin University, The Royal Melbourne Hospital, Deakin University, The University of Melbourne, 300 Grattan Street, Parkville, VIC 3050 Australia; 14 Neurology, Melbourne Brain Centre, The Royal Melbourne Hospital, 300 Grattan Street, Parkville, 3050 VIC Australia

**Keywords:** Acute ischaemic stroke, Mild stroke, CT perfusion, Tmax + 6s, Large vessel occlusion, Reperfusion therapy

## Abstract

**Background:**

Guidelines generally advise against reperfusion therapy in patients with mild stroke (NIHSS ≤ 5) and non-disabling symptoms. However, stroke severity can fluctuate, and clinical scores may not fully capture tissue at risk. Reliance on non-contrast CT (NCCT), potentially missing perfusion deficits or large vessel occlusions (LVOs). Advanced imaging—including CT angiography (CTA) and CT perfusion (CTP)—can reveal significant hypoperfusion in otherwise mild presentations. This study aimed to quantify the proportion of increased tissue-at-risk volumes (Tmax + 6s ≥ 15 mL) in patients with mild acute ischaemic stroke and identify associated factors and outcomes.

**Methods:**

We included consecutive AIS patients within 24 h of onset from multicentre stroke registries in Australia and Indonesia. Only those with baseline NCCT, CTA, and CTP were analysed. Patients were stratified into NIHSS ≤ 5 and > 5. Tissue-at-risk was defined as Tmax + 6s ≥ 15 mL. Clinical, imaging, and outcome data were compared, and predictors of poor functional outcome (mRS 3–6 at 90-day) were assessed.

**Results:**

Of 655 patients, 314 had NIHSS ≤ 5. Among these, 22.9% exhibited Tmax + 6s ≥ 15 mL, indicating significant hypoperfusion. This subgroup had worse 90-day outcomes (26.4% mRS 3–6 vs. 9.5%, *p* < 0.001). Tmax + 6s ≥ 15 mL, hypertension, and LVO were independently associated with poor outcome (adjusted ORs: 2.51, 3.15, and 2.74 respectively). ROC analysis demonstrated moderate discrimination of Tmax + 6s volume for poor functional outcome.

**Conclusions:**

A substantial proportion of mild stroke patients harbour treatable perfusion deficits. CT perfusion provides essential prognostic information beyond clinical severity, supporting its role in guiding therapeutic decisions—even in low NIHSS presentations where standard imaging may otherwise overlook tissue at risk.

**Supplementary Information:**

The online version contains supplementary material available at 10.1186/s42466-025-00442-8.

## Introduction

Current acute ischaemic stroke (AIS) guidelines generally recommend against administering reperfusion therapy to patients with mild, non-disabling symptoms, typically defined by National Institutes of Health Stroke Scale (NIHSS) scores ≤ 5 [1]. While the NIHSS is widely used for initial assessment, it does not fully capture the clinical spectrum of stroke impact, particularly in cases involving visual or attentional deficits such as hemianopia or neglect [2]. The NIHSS’s emphasis on motor function may result in low scores that underestimate clinical severity, leading to under-recognition of other clinically significant impairments [2, 3]. This underestimation can lead to inappropriate exclusion from reperfusion therapy, potentially resulting in poor outcomes as reflected by higher modified Rankin Scale (mRS) scores—even among patients with apparently mild strokes [2].

The NIHSS does not reflect individual variations in cerebral haemodynamics, which can influence clinical presentation and complicate consistent scoring [2, 4]. Inter-rater variability further limits its reliability as a sole decision-making tool [4–6]. These limitations highlight the importance of incorporating imaging-based modalities to improve the precision of stroke evaluation and guide therapeutic decision-making [4–6].

Emerging evidence suggests that patients with CT perfusion (CTP) parameters showing Tmax + 6 s volumes ≥ 15 mL may benefit from reperfusion therapy [7–10]. This includes patients with NIHSS ≤ 5, who may still harbour substantial tissue at risk. Identifying such patients is clinically important, as perfusion-based selection may help prevent infarct progression and improve outcomes [7, 10]. However, current guidelines have not fully integrated perfusion criteria, potentially leading to under-treatment of this high-risk subgroup [1, 10].

To address this gap, we conducted a retrospective study analysing multimodal CT imaging data from Australian and Indonesian stroke registries. We examined the relationship between NIHSS scores and perfusion-defined tissue at risk (Tmax + 6 s ≥ 15 mL), with a particular focus on patients with NIHSS ≤ 5. We hypothesized that a meaningful proportion of clinically mild strokes would demonstrate substantial tissue at risk, indicating treatment opportunities that are currently overlooked.

## Methods

We analysed multimodal CT imaging data collected between February 2018 and February 2020 from acute ischaemic stroke (AIS) cases in Australia and Indonesia. Imaging included non-contrast CT (NCCT), CT perfusion (CTP), and CT angiography (CTA). Cases were included if they presented within 24 h of symptom onset and had complete multimodal CT data available for analysis. Cases with haemorrhagic stroke or incomplete multimodal CT imaging were excluded.

All imaging was performed on admission using standardised institutional protocols. CT perfusion datasets were processed using RAPID software (versions 5.0.2 and 5.0.4), which automatically generates maps of cerebral blood flow, cerebral blood volume, mean transit time, and Tmax delay. Quantitative perfusion lesion volumes were calculated, and tissue at risk was defined as regions with Tmax + 6 s volume exceeding the pre-specified threshold.

CT angiography was used to determine large vessel occlusion (LVO), defined as the absence of contrast opacification in a major intracranial artery, including the intracranial internal carotid artery (ICA), M1 or M2 segments of the middle cerebral artery (MCA), basilar artery (BA), or vertebral artery (VA).

The study was approved by the institutional ethics review boards of the participating hospitals and conducted in accordance with the principles of the Declaration of Helsinki. Reporting followed the Strengthening the Reporting of Observational Studies in Epidemiology (STROBE) guidelines.

### Brain imaging acquisition protocols

All CT perfusion (CTP) datasets were processed using RAPID automated software (iSchemaView/Stanford University; version 5.0.2 for Jakarta, 5.0.4 for Geelong). Processing followed standardised protocols that generated parametric maps of cerebral blood flow, cerebral blood volume, mean transit time and Tmax delay. Quantitative volumes were computed for each Tmax + 6 s threshold, and tissue at risk was defined as regions exceeding this delay threshold. Detailed imaging parameters are provided in the Supplementary Material Imaging.

### Stroke imaging registry

We developed a prospective, bi-national registry for acute ischaemic stroke (AIS) patients over time, incorporating data from Australia’s University Hospital Geelong (Victoria) and Jakarta, National Brain Centre hospital. Both locations enrolled AIS patients aged 18 and above who arrived at the hospital within 24 h of symptom onset, excluding those with intracerebral haemorrhage. Collected data encompassed demographics, NIHSS-assessed stroke severity, vascular risk factors, and timing of events. Outcomes were measured using the Modified Rankin Scale (mRS) at a 3-month follow-up. At manuscript preparation, the registry included 655 AIS patients, complete with imaging data such as non-contrast CT, CT perfusion, CT angiography, and follow-up MRI with DWI/ADC. The Jakarta site accounted for 418 patients, while Geelong contributed 237.

### Large vessel occlusion assessment

All patients underwent CT angiography (CTA) to evaluate intracranial vessel patency. Large vessel occlusion (LVO) was defined by absence of contrast opacification in a major intracranial artery and was categorized by anatomical location: intracranial internal carotid artery (ICA), M1 or M2 segments of the middle cerebral artery (MCA), basilar artery or vertebral artery.

### Statistical analyses

Continuous variables with normal distribution are reported as mean ± standard deviation and compared using independent t-tests. Categorical variables are presented as counts and percentages and analysed using the chi-square test or Fisher’s exact test, as appropriate. Continuous variables not normally distributed are reported as median (interquartile range [IQR]) and compared using the Mann-Whitney U test. For comparisons involving more than two groups of non-normally distributed data, the Kruskal-Wallis test was employed. Tissue-at-risk assessment was based on Tmax + 6 s volumes determined by RAPID software for standardised, algorithm-based quantification. The relationship between NIHSS strata and tissue-at-risk volumes was also evaluated using the Kruskal-Wallis test.

## Results

Baseline characteristics of the study cohort (*n* = 655) are summarised in Table 1, stratified by NIHSS ≤ 5 (*n* = 314) and NIHSS > 5 (*n* = 341). No significant differences were observed in age, sex, or vascular risk factors between the two groups, except for a higher prevalence of smoking in the NIHSS > 5 group (*p* = 0.043). Large vessel occlusion (LVO) was present in 163 of 655 patients (24.9), including 36 of 314 (11.5%) with NIHSS ≤ 5 and 127 of 341 (37.2%) with NIHSS > 5.

**Table 1:** Baseline characteristics stratified by NIHSS ≤ 5 and > 5

Variables	NIHSS ≤ 5 (*n* = 314)	NIHSS > 5 (*n* = 341)	*P* value
Age ^1^	65 ± 14	63 ± 13	0.076
Gender^2^			
Male^2^	209 (66.6%)	232 (68%)	0.750
Risk Factor^2^			
Hypercholesterolemia	81 (25.8%)	88 (25.8%)	1.000
Hypertension	223 (71%)	252 (73.9%)	0.461
Atrial Fibrillation	40 (12.7%)	50 (14.7%)	0.548
Diabetes Mellitus	85 (27.1%)	100 (29.3%)	0.580
Smoking^2^	66 (21%)	96 (28.2%)	0.043*
Previous stroke^2^	84 (26.8%)	90 (26.4%)	0.988
Onset to scanning time (min) ^3^	175 (107–343)	125 (91–335)	0.126
Large Vessel Occlusion	36 (11.5%)	127 (37.2%)	< 0.001*
MCA1	14 (4.5%)	80 (23.5%)	< 0.001*
MCA2	11 (3.5%)	28 (8.2%)	0.017*
ACA	1 (0.3%)	12 (3.5%)	0.008*
PCA	5 (1.6%)	7 (2.1%)	0.883
Basilar	2 (0.6%)	1 (0.3%)	0.610
Vertebral	5 (1.6%)	2 (0.6%)	0.269
ICA	4 (1.3%)	18 (5.3%)	0.009*

^1^ Analysis numerical variable with normal distribution used independent t-test. data presented with mean ± standard deviation

^2^ Analysis categorical variable by using the frequency and percentages used chi-square test/ fisher exact test

^3^ Analysis numerical variable with non-normal distribution used Mann-Whitney test. data presented with median (IQR)

*Statistically significant

Baseline demographics were largely comparable, though large vessel occlusion and smoking were significantly more prevalent in patients with NIHSS > 5

Among patients with NIHSS ≤ 5, 72 of 314 (22.9%) exhibited a Tmax + 6 s volume ≥ 15 mL, indicating haemodynamically significant hypoperfusion. In comparison, 188 of 341 patients (55.1%) in the NIHSS > 5 group reached the same threshold (Table 2). Perfusion imaging revealed marked differences in ischaemic burden between groups. The median Tmax + 6 s volume in the NIHSS ≤ 5 group was 0 mL (IQR 0–11.25), whereas the NIHSS > 5 group had a median of 28 mL (IQR 0–116.5; *p* < 0.001). Most patients with mild stroke (74.8%) demonstrated perfusion volumes of 0–10 mL, while 14.0% had lesions exceeding 31 mL (Supplementary material see Table S1 and Figure S1).

**Table 2:** Distribution of Tmax + 6s volumes Stratified by Stroke Severity NIHSS ≤ 5 versus > 5

Tmax + 6s volume (mL)	NIHSS ≤ 5 (*n* = 314)	NIHSS > 5 (*n* = 341)	*P*-Value
Tmax + 6s volume ≥ 15	72 (22.9%)	188 (55.1%)	< 0.001*
Tmax + 6s volume < 15	242 (77.1%)	153 (44.9%)	
Tmax + 6s volume 0–10 mL	235 (74.8%)	143 (41.9%)	< 0.001*
Tmax + 6s volume 11–20 mL	23 (7.3%)	15 (4.4%)	
Tmax + 6s volume 21–30 mL	12 (3.8%)	17 (5.0%)	
Tmax + 6s volume ≥ 31 mL	44 (14.0%)	166 (48.7%)	

Patients with NIHSS > 5 more frequently exhibit higher Tmax+6s volume, while 22.9% of mild stroke still harboured ≥ 15 mL tissue at risk

**Fig. 1:**
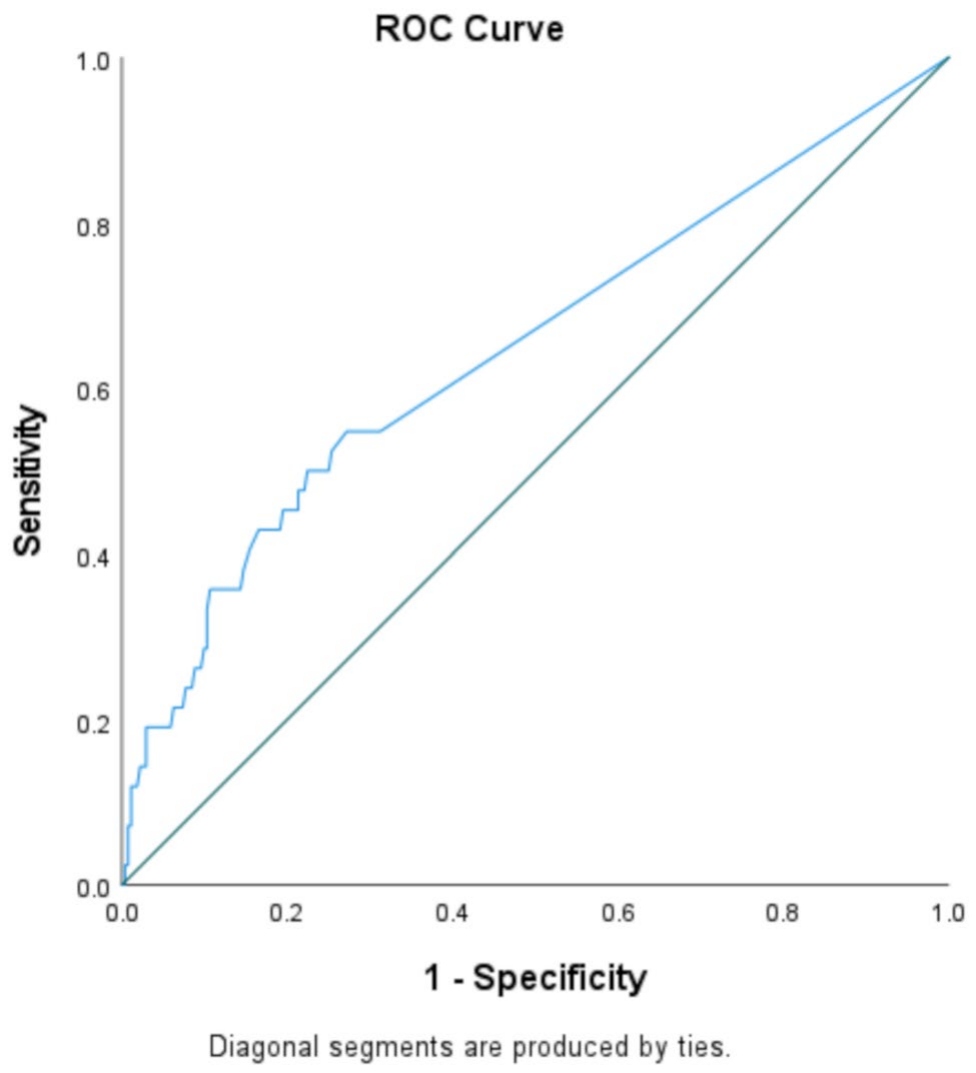
ROC Curve of Tmax + 6s Volume for Predicting Poor Functional Outcome (mRS 3-6) in NIHSS ≤5 The Receiver Operating Characteristic (ROC) curve illustrates the performance of Tmax +6s volume in identifying patients with poor functional outcomes among those presenting with mild stroke (NIHSS ≤5). A Tmax +6s volume threshold ≥ 15 ml demonstrated modest sensitivity but high specificity (80.5 %), highlighting its value as a selective marker of clinically significant tissue at risk. In this mild-stroke cohort, where clinical signs alone may under-estimate ischemic burden, Tmax +6s volume provides complementary prognostic information to guide individualised treatment decisions

Receiver operating characteristic (ROC) analysis in the NIHSS ≤ 5 subgroup showed that a Tmax + 6 s threshold ≥ 15 mL predicted poor 90-day functional outcomes (mRS 3–6) with 45.2% sensitivity and 80.5% specificity (Fig. 1). Increasing the threshold improved specificity but reduced sensitivity, supporting 15 mL as a clinically relevant cut-off for identifying perfusion-defined tissue at risk in mild stroke (see Supplementary material Table S2).

Factors independently associated with Tmax + 6 s ≥ 15 mL in the NIHSS ≤ 5 group included atrial fibrillation (OR 4.83, 95% CI 2.42–9.65), higher baseline NIHSS (OR 1.21 per point), and LVO (OR 15.53), particularly M1 (OR 53.10) and ICA (OR 20.85) occlusions (Table 3). Other vascular risk factors, including hypertension, diabetes, and smoking, were not significantly correlated with larger perfusion volumes.

**Table 3:** Factors associated with Tmax + 6s (mL) ≥ 15 ml in patients NIHSS Score ≤ 5

Variables	Tmax + 6s (mL)		*P*-value	OR (95% CI)^4^
	< 15 mL (*n* = 242)	≥ 15 mL (*n* = 72)		
Age ^1^	71 ± 14	73 ± 13	0.713	1.00 (0.98–1.02)
Gender^2^				
Male	157 (64.9%)	52 (72.2%)	0.309	1.41 (0.79–2.51)
Female	85 (35.1%)	20 (27.8%)		
Risk Factor^2^				
Hypercholesterolemia	66 (27.3%)	15 (20.8%)	0.346	0.70 (0.37–1.33)
Hypertension	173 (71.5%)	50 (69.4%)	0.851	0.91 (0.51–1.61)
Atrial Fibrillation	19 (7.9%)	21 (29.2%)	< 0.001*	4.83 (2.42–9.65)
Diabetes Mellitus	67 (27.7%)	18 (25%)	0.765	0.87 (0.48–1.59)
Smoking	49 (20.2%)	17 (23.6%)	0.653	1.22 (0.65–2.28)
Previous stroke	59 (24.4%)	25 (34.7%)	0.112	1.65 (0.94–2.91)
NIHSS score^3^	2 (0–5)	2.5 (0–5)	< 0.005*	1.21 (1.17–1.26)
Onset to scanning time (min) ^3^	175 (18–1673)	170.5 (23–1155)	0.753	1.00 (1.00 − 1.01)
LVO ^2^	9 (3.7%)	27 (37.5%)	< 0.001*	15.53 (6.85–35.24)
MCA1	1 (0.4%)	13 (18.1%)	< 0.001*	53.10 (6.81–414.05)
MCA2	3 (1.2%)	8 (11.1%)	< 0.001*	9.96 (2.57–38.62)
ACA	0 (0%)	1 (1.4%)	0.229	-
PCA	0 (0%)	5 (6.9%)	< 0.001	-
Basilar	0 (0%)	2 (2.8%)	0.052	-
Vertebral	4 (1.7%)	1 (1.4%)	1.000	0.84 (0.09–7.62)
ICA	0 (0%)	4 (5.6%)	0.003	-

^1^ Statistical analysis used independent t-test for numerical normal distribution

^2^ Statistical analysis used chi-square test

^3^ Statistical analysis used mann-whitney test for numerical non-normal distribution, data presented with median (IQR)

^4^ Odds Ratio (OR) calculated for categorical variable

^*^ Statiscally significant

In patients with NIHSS ≤ 5, Tmax + 6s volume ≥ 15 was significantly associated with atrial fibrillation, in particular MCA M1 segment

Among patients with NIHSS ≤ 5, those with Tmax + 6 s ≥ 15 mL had poor 90-day functional outcomes more frequently, with a median mRS of 1 (IQR 0–3) compared to 0 (IQR 0–1) in those with < 15 mL. Functional dependence (mRS 3–6) occurred in 26.4% versus 9.5%, respectively (*p* < 0.001; Supplementary Table S3). Univariate analyses identified Tmax + 6 s ≥ 15 mL (OR 3.41), LVO (OR 4.13), and hypertension (OR 2.73) as significant predictors of poor 90-day functional outcome (Supplementary Table S4). Multivariate logistic regression confirmed that Tmax + 6 s ≥ 15 mL (aOR 2.51), hypertension (aOR 3.15), and LVO (aOR 2.74) were independent predictors of poor functional outcomes at 90 days (Table 4).

**Table 4:** Multivariate analysis of predictor of poor 90-day outcome (mRS 3–6) in patients NIHSS ≤ 5

Variables	Crude Multivariate			Adjusted Multivariate		
	cOR	95%CI	*P* Value	aOR	95%CI	*p*-value
Tmax + 6s volume ≥ 15 ml	2.69	1.18–6.13	0.018	2.51	1.15–5.49	0.021*
Gender	0.84	0.40–1.78	0.648			
Age	1.01	0.98–1.04	0.393			
Hypercholesterolemia	0.61	0.25–1.44	0.257			
Hypertension	3.14	1.20–8.23	0.020*	3.15	1.23–8.02	0.016*
Atrial Fibrillation	0.90	0.33–2.48	0.837			
Diabetes Mellitus	1.69	0.79–3.63	0.175			
Smoking	0.57	0.20–1.60	0.285			
Previous History of Stroke	0.84	0.38–1.87	0.667			
Large Vessel Occlusion	2.99	1.15–7.78	0.025*	2.74	1.09–6.86	0.031*

Note: Multivariate analysis used Multiple Binary Logistics involving socio-demographic and risk factors variable

*Statistically significant (*p* value < 0.05)

Multivariate analysis confirmed that Tmax + 6s volume ≥ 15 mL (aOR 2.51), hypertension(aOR3.15) and LVO (aOR 2.74) were independently associated with poor functional outcomes at 90 days among patients with NIHSS ≤ 5

**Fig. 2:**
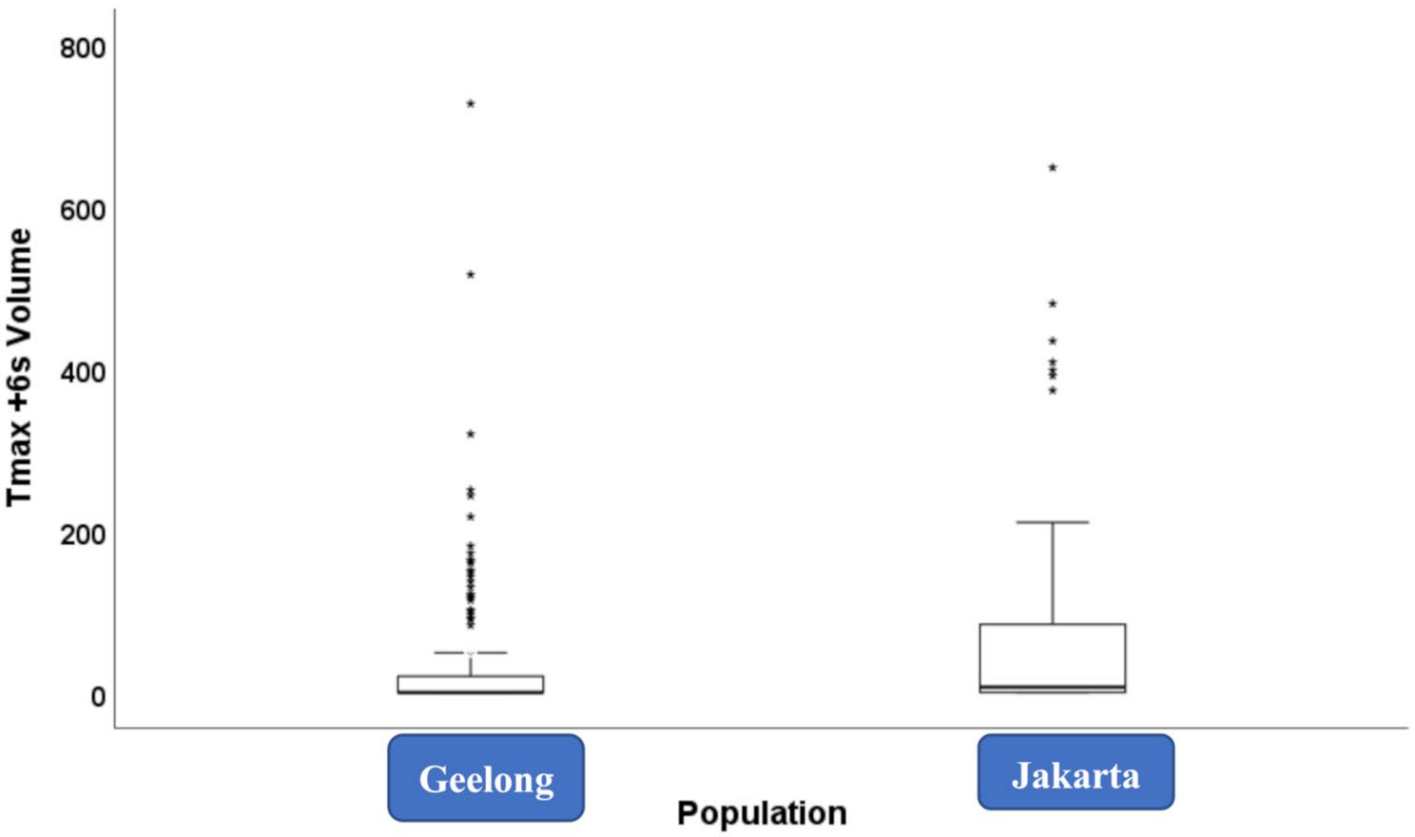
Box plot showing the distribution of Tmax + 6 s volumes in acute ischemic stroke patients, stratified by study population (Geelong vs. Jakarta) The Jakarta cohort demonstrated a higher median volume, a wider interquartile range, and more extreme outliers, reflecting a greater burden of tissue at risk, consistent with delayed presentation or more severe perfusion deficits

**Fig. 3:**
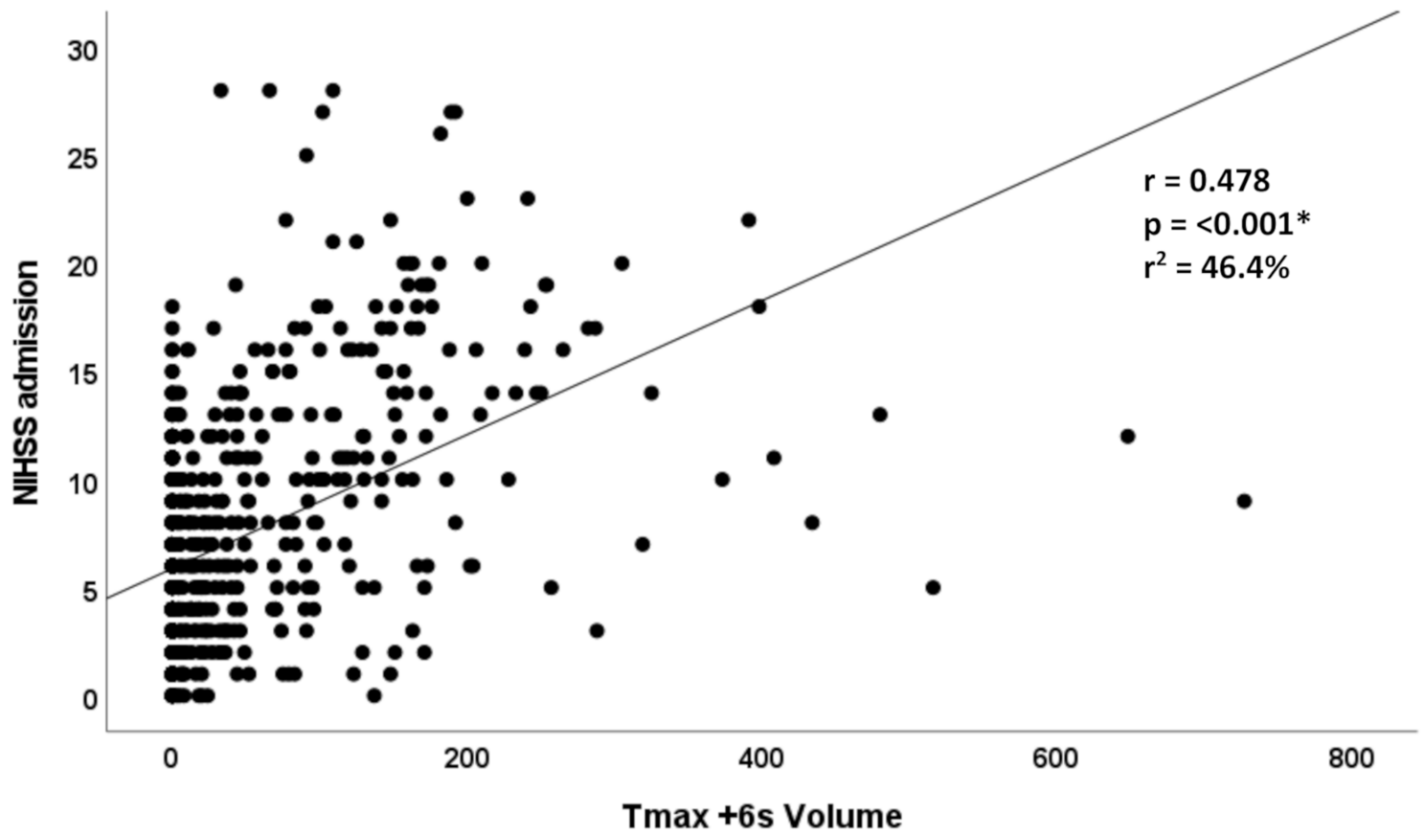
Scatter plot illustrating the correlation between Tmax + 6 s volume and NIHSS score at admission Larger perfusion deficits were generally associated with higher stroke severity, showing a positive trend between ischemic tissue-at-risk volume and initial clinical presentation

Finally, stratified analysis demonstrated a progressive increase in Tmax + 6 s volumes across mRS categories, underscoring the prognostic relevance of perfusion-defined tissue at risk even in initially mild stroke presentations (Figs. 2 and 3).

## Discussion

Our findings demonstrated that 22.9% of patients with low NIHSS scores (≤ 5)—who are typically excluded from reperfusion therapy [1, 10]—harboured clinically significant tissue at risk, defined as a Tmax + 6 s volume ≥ 15 mL. In contrast to some prior reports suggesting that cardioembolic strokes may not always present with large early perfusion lesions [11, 12], atrial fibrillation in our cohort was significantly associated with larger perfusion deficits (Tmax + 6 s ≥ 15 mL), consistent with a cardioembolic aetiology and a tendency to produce extensive hypoperfused territories in the absence of early recanalisation. This distinction is clinically important, as CT perfusion parameters can reveal tissue at risk even in patients with mild neurological deficits, allowing for early identification of those vulnerable to neurological deterioration and poor functional outcomes [13–15].

The NIHSS remains a cornerstone of acute ischaemic stroke (AIS) assessment but has important limitations, particularly in mild presentations [16]. It is weighted toward anterior circulation deficits and may under-represent posterior circulation signs such as truncal ataxia, nystagmus, visual field deficits, or tandem gait disturbances [2, 6, 17–19]. Consequently, patients with clinically mild strokes can harbour substantial perfusion-defined tissue at risk that is not captured by NIHSS alone. Our findings support the integration of CT perfusion parameters, particularly Tmax + 6 s volume, to complement clinical assessment and enhance triage for low NIHSS patients in the acute ischaemic stroke setting.

The definition of tissue at risk using perfusion maps is not fully standardised, as studies use different thresholds and parameters (e.g., Tmax, CBV, CBF). Scanner hardware, acquisition protocols, and post-processing software influence volumetric estimates. In this study, RAPID software was used to minimize variability, as it is validated in multiple multicentre stroke trials. However, results may still vary across platforms and settings, reflecting the broader challenge of standardizing perfusion imaging in acute ischaemic stroke [20].

We selected Tmax + 6 s ≥ 15 mL as our perfusion threshold based on prior validation in the DEFUSE 3, EXTEND, and CATCH studies [7–9, 13, 14, 21]. These studies demonstrated that lesions with a Tmax + 6 s delay often represent critically hypoperfused but potentially salvageable tissue and that quantitative perfusion mapping can predict infarct growth in the absence of timely reperfusion. We confirmed that Tmax + 6 s volume correlates with NIHSS in our cohort, and our multivariable regression accounted for age, hypertension, and LVO status (Table 4), supporting the validity of this threshold. In our study, Tmax + 6 s ≥ 15 mL was independently associated with poor 90-day functional outcome (adjusted odds ratio [aOR] 2.51). ROC analysis demonstrated high specificity (80.5%) for predicting functional dependence, reinforcing that this threshold is a clinically meaningful cut-off for identifying patients with mild stroke who may benefit from reperfusion therapy.

Importantly, 22.9% of low-NIHSS patients harboured perfusion-defined tissue at risk (Tmax + 6 s ≥ 15 mL), identifying the subgroup most vulnerable to early neurological deterioration (END). Only 13.4% developed poor 90-day outcomes (mRS 3–6), reflecting the gap between imaging-defined risk and clinical disability, as some patients recover with robust collaterals or early reperfusion. This aligns with prior reports of 20–30% END in mild LVO strokes [11]. These findings highlight the prognostic value of CTP in mild strokes, enabling early recognition of high-risk patients who may be missed by NIHSS-based triage and supporting targeted monitoring and treatment decisions [22].

Our stratified analysis showed a progressive increase in Tmax + 6 s volumes across mRS categories, emphasising the prognostic value of perfusion-defined tissue at risk. Approximately 20–30% of patients with NIHSS ≤ 5 experience early neurological deterioration (END), particularly when LVO or perfusion mismatch is present [11, 12, 15, 20, 23]. In our study, 11.5% of NIHSS ≤ 5 patients had a large vessel occlusion (LVO), most commonly in the M1 segment, supporting the observation that large ischaemic volumes with low NIHSS often correspond to underlying LVO. LVO and hypertension were independent predictors of poor 90-day outcomes, highlighting the importance of integrating vascular imaging and CT perfusion parameters in clinical decision-making. highlighting the importance of integrating vascular imaging and CT perfusion parameters in clinical decision-making. Importantly, these imaging findings are directly linked to **clinically meaningful patient outcomes**, demonstrating that tissue-at-risk volumes predict functional dependence rather than serving as imaging surrogates alone. This addresses the concern that “*images rather than patients are being treated*,” ensuring that perfusion imaging complements patient-centred decision-making. In addition, long term follow-up studies, such as Schabitz et al.l have shown that younger age and lower NIHSS are predictors of good functional outcome and quality of life at 2.5 years post stroke, reinforcing our findings via CT perfusion may benefit from more advanced imaging and potentially earlier reperfusion therapy [24].

These findings have direct implications for emergency stroke triage. Conventional workflows often rely on NIHSS thresholds to guide thrombolysis and thrombectomy eligibility, potentially overlooking patients with low scores but significant tissue at risk. Integrating CT perfusion early into the acute ischaemic stroke workflow—either immediately upon hospital arrival or via prehospital imaging pathways—could allow for precise selection of low NIHSS patients who may benefit from reperfusion therapy [25–27] (Supplementary Material Acute Workflow). This imaging-guided strategy is supported by recent trials, including TEMPO 2 and CATCH, which highlight the limitations of NIHSS-only selection and the added value of perfusion-based assessment [13, 14].

Our study has several limitations. Its retrospective design introduces potential selection bias, as only patients with available CT perfusion were included. While Tmax + 6 s volume is a validated perfusion parameter, it can overestimate tissue at risk in the presence of robust collateral flow, and perfusion thresholds are not fully standardised across software platforms [8, 28]. Nonetheless, the use of RAPID software—widely implemented in multicentre trials—enhances reproducibility and clinical applicability. Future prospective studies with standardised imaging protocols and functional follow-up are warranted to determine whether early integration of perfusion imaging can expand treatment opportunities for this under-recognised subgroup of patients.

In summary, CT perfusion could identify treatment opportunities in patients with mild ischaemic stroke (NIHSS ≤ 5). Approximately one in five patients exhibited significant tissue-at-risk volumes (Tmax + 6 s ≥ 15 mL), reliably delineating tissue at risk and predicting poor functional outcomes. These findings support the integration of CT perfusion into early acute stroke workflows to guide timely intervention and close the treatment gap in this population.

## Supplementary Information

Below is the link to the electronic supplementary material.

Supplementary Material 1

## Data Availability

Data are not publicly available due to patient confidentiality but may be shared on reasonable request to the corresponding author.

## Abbreviations

CTP: Computed Tomography Perfusion
END: Early Neurological Deterioration
ICA: Internal Carotid Artery
IQR: Interquartile Range
LVO: Large Vessel Occlusion
MCA: Middle Cerebral Artery
mRS: Modified Rankin Scale
NCCT: Non-Contrast Computed Tomography
NIHSS: National Institutes of Health Stroke Scale
aOR: Adjusted Odds Ratio
ROC: Receiver Operating Characteristic
Tmax + 6s: Time-to-maximum of the residue function plus 6 s
