# Supplementary material for: CT perfusion identified potential treatment opportunities in one in five mild strokes
Source: Neurol Res Pract. 2025 Nov 7;7(1):85. doi: 10.1186/s42466-025-00442-8 (PMC12595888; doi:10.1186/s42466-025-00442-8)
Supplement: Supplementary file 1 — Supplementary Material 1 [file 42466_2025_442_MOESM1_ESM.docx]

**Table S1. CT Perfusion Parameters Stratified by NIHSS ≤5 versus >5**

| Variables | Total (n = 655) | NIHSS ≤5  (n=314) | NIHSS >5  (n=341) | P-Value |
| --- | --- | --- | --- | --- |
| rCBF <30% (mL) median (IQR) | 0 (0 – 3) | 0 (0 – 0) | 0 (0 – 15.5) | <0.001* |
| Tmax+6s (mL) median (IQR) | 3 (0 – 52) | 0 (0 - 11.25) | 28 (0 - 116.5) | <0.001* |

^1^ Analysis numerical variable with non-normal distribution used Mann-Whitney test. data presented by median (interquartile range). *Statistically significant

**Median Tmax+6s and rCBF<30% volumes were significant lower in the NIHSS ≤5 group, indicating reduced perfusion deficits in milder stroke.**

**Table S2. Sensitivity Analysis of Tmax + 6s Volume Threshold in Patients with NIHSS ≤5**

| **Tmax + 6s volume** | **NIHSS ≤5** |
| --- | --- |
| Tmax + 6s volume ≤5 mL | 223/357 (62.5%) |
| Tmax + 6s volume ≤10 mL | 235/378 (62.2%) |
| Tmax + 6s volume ≤15 mL | 244/398 (61.3%) |
| Tmax + 6s volume ≤20 mL | 258/416 (62.0%) |
| Tmax + 6s volume ≤25 mL | 266/433 (61.4%) |
| Tmax + 6s volume ≤30 mL | 270/445 (60.7%) |

**A Tmax + 6s ≤ 15 mL captured 61.3 % NIHSS ≤ 5, supporting its utility for stratifying low-severity mild stroke.**

**Table S3. Ninety-Day mRS Outcomes in Patients with NIHSS ≤ 5 Stratified by Tmax + 6s Volume**

| Variables | Tmax +6s (mL) | | P-value |
| --- | --- | --- | --- |
|  | <15 mL  (n=242) | ≥15 mL  (n=72) |  |
| mRS score^3^ | 0 (0 – 1) | 1 (0 – 3) | <0.001* |
| mRS Category^2^ |  |  |  |
| Good (MRS 0 – 2) | 219 (90.5%) | 53 (73.6%) | <0.001* |
| Poor (MRS 3 – 6) | 23 (9.5%) | 19 (26.4%) |  |

^2^ Statistical analysis used chi-square test

^3^ Statistical analysis used mann-whitney test for numerical non-normal distribution, data presented with median (IQR)

^*^ Statistically significant

**Among NIHSS ≤5 patients, those with Tmax+6s volume ≥15 had significantly worse 90-day outcomes, with higher median mRS scores compared to those with Tmax+6s volume<15 mL.**

**Table S4. Predictors of Poor 90-Day Functional Outcome (mRS 3-6) in Patients**

**with NIHSS ≤5**

| Variables | MRS at 90 Days | | P-value | OR (95% CI)^4^ |
| --- | --- | --- | --- | --- |
|  | Good  (n=272) | Poor  (n=42) |  |  |
| Age ^1^ | 64 ± 14 | 68 ± 12 | 0.065 | 1.02 (1.00 – 1.05) |
| Gender^2^ |  |  |  |  |
| Male | 183 (87.6%) | 26 (12.4%) | 0.609 | 0.79 (0.40 – 1.55) |
| Female | 89 (84.8%) | 16 (15.2%) |  |  |
| Risk Factor^2^ |  |  |  |  |
| Hypercholesterolemia | 72 (88.9%) | 9 (11.1%) | 0.613 | 0.76 (0.35 – 1.66) |
| Hypertension | 187 (83.9%) | 36 (16.1%) | 0.038 | 2.73 (1.11 – 6.72) |
| Atrial Fibrillation | 32 (80.0%) | 8 (20.0%) | 0.285 | 1.77 (0.75 – 4.15) |
| Diabetes Mellitus | 71 (83.5%) | 14 (16.5%) | 0.427 | 1.42 (0.71 – 2.84) |
| Smoking | 60 (90.9%) | 6 (9.1%) | 0.343 | 0.59 (0.24 – 1.46) |
| Previous stroke | 73 (86.9%) | 11 (13.1%) | 1.000 | 0.97 (0.46 – 2.02) |
| Tmax +6s (mL) ^2^ |  |  |  |  |
| <15 mL | 219 (90.5%) | 23 (9.5%) | <0.001* | 3.41 (1.73 – 6.72) |
| ≥15 mL | 53 (73.6%) | 19 (26.4%) |  |  |
| LVO^2^ | 24 (66.7%) | 12 (33.3%) | <0.001* | 4.13 (1.88 – 9.11) |
| MCA1 | 8 (57.1%) | 6 (42.9%) | 0.005* | 5.50 (1.81 – 16.76) |
| MCA2 | 7 (63.6%) | 4 (36.4%) | 0.045* | 3.99 (1.11 – 14.26) |
| ACA | 1 (100%) | 0 (0%) | 1.000 | - |
| PCA | 3 (60.0%) | 2 (40.0%) | 0.134 | 4.48 (0.73 – 27.66) |
| Basilar | 1 (50.0%) | 1 (50.0%) | 0.250 | 6.61 (0.41 – 107.74) |
| Vertebral | 5 (100%) | 0 (0%) | 1.000 | - |
| ICA | 1 (25.0%) | 3 (75.0%) | 0.008* | 20.85 (2.12 – 205.43) |

^1^ Statistical analysis used independent t-test for numerical normal distribution

^2^ Statistical analysis used chi-square test/ fisher exact test

^4^ Odd Ratio (OR) calculated for categorical variable

^*^Statiscally significant

**In patient with NIHSS ≤5, Tmax +6s volume ≥15 mL (OR 3.41), LVO (OR.4.13), hypertension (OR 2.73) were independently associated with poor functional outcome at 90 days (mRS 3-6)**


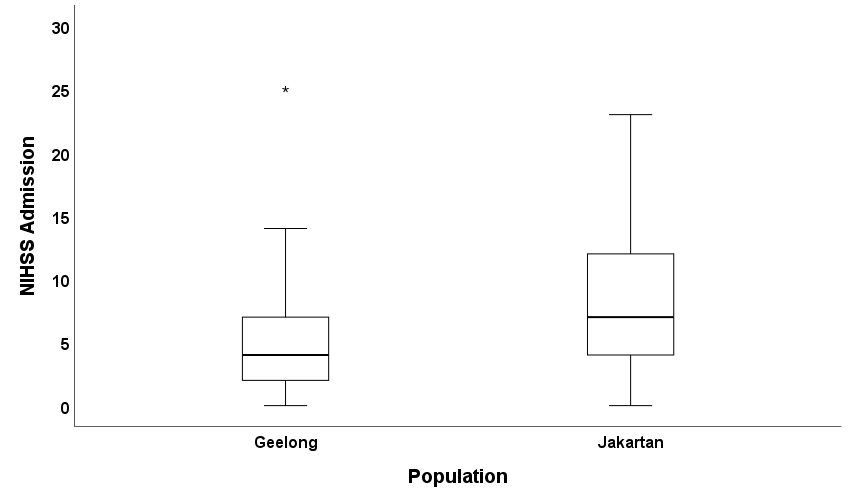
 **Figure S1. Box plot comparing NIHSS scores at admission between the Geelong and Jakarta cohorts.**

**Jakarta**

**Geelong**

The Jakarta group exhibited a higher median NIHSS and a wider interquartile range, indicating more severe stroke presentations at the time of hospital admission.

**Imaging Protocols**

**Imaging in National Brain Centre, Jakarta, Indonesia**

**CTP scanning Protocol**

A whole-brain non-contrast CT (NCCT) was performed in one rotation (detector width 8–16 cm system DAS; thickness 5–10 mm), followed by whole-brain CTP and CTA. Image acquisition was initiated after intravenous injection of 90 mL of Ultravist 370 (Bayer HealthCare) at 6 mL/s, followed by a 30 mL saline flush at the same rate. Each image was reconstructed with a slice thickness of 5 mm, and 35 images were acquired every 1.5 seconds (total acquisition time = 53 seconds). The perfusion coverage was 8 cm, 80 kVp, and 100 mAs.

**CTP analysis**

CTP image data were auto-sent to a research version of RAPID automated software (Stanford University and iSchemaView) for post-processing. Estimation of ischaemic core (relative cerebral blood flow), penumbra (Tmax), relative cerebral blood volume, mismatch volume, mismatch ratio (Tmax/CBF), mean transit time, time to peak, motion estimate curves, arterial input function from the middle cerebral artery (AIF), and venous output function from the superior sagittal sinus (VOF) were automatically generated and measured by RAPID.

**CTA Reference Standard**

CTA coverage: Multiplanar reconstruction (MPR) images were generated in several planes with a slice thickness of 0.9 mm, using data acquired from a Philips iCT 256-slice scanner. Acquisition parameters were 120 kVp, radiation dose 31.9 mGy, and DLP 801.2 mGy·cm.

**MRI Reference Standard**

MRI was performed on a 3 T Siemens Skyra scanner. Diffusion-weighted imaging (DWI) was acquired using b-values of 0 s/mm² and 1000 s/mm² with an axial isotropic DWI sequence (slice thickness 4 mm; field-of-view [FOV] 220 mm). The raw MRI data were transferred to a research version of RAPID automated software (Stanford University and iSchemaView) for post-processing and apparent diffusion coefficient (ADC) volume measurement to determine final ischaemic core volume.

**Imaging in Geelong University Hospital, Australia**

A whole-brain non-contrast CT (NCCT) was performed in a single rotation (detector width 8–16 cm; system Data Acquisition System (DAS); slice thickness 5–10 mm), followed by CTP and CTA.

**CTP Scanning Protocol**

A whole-brain non-contrast CT (NCCT) was performed in a single rotation (detector width 8–16 cm; system Data Acquisition System [DAS]; slice thickness 5–10 mm), followed by whole-brain CTP and CTA. Image acquisition was initiated after intravenous injection of 125 mL of Ultravist 370 (Bayer HealthCare) at 5 mL/s, followed by a 30 mL saline flush at the same rate. Each image was reconstructed with a slice thickness of 5 mm, and 35 images were acquired every 2 seconds (total acquisition time = 70 seconds). The perfusion coverage was 8 cm, with parameters of 80 kVp and 80 mAs.

**CTA Reference Standard**

CTA coverage: Multiplanar reconstruction (MPR) images were generated in multiple planes with a slice thickness of 0.8 mm, using data acquired from a Philips iCT 256-slice scanner. Acquisition parameters were 100 kVp with a radiation dose of 80 mGy.

**MRI Reference Standard**

MRI was performed using Siemens 1.5 T Avanto and 3 T Prisma Fit scanners. The stroke MRI protocol included diffusion-weighted imaging (DWI) with b-values of 0 s/mm² and 1000 s/mm². An axial isotropic DWI sequence (slice thickness 3 mm; field of view [FOV] 220 mm) was used. DWI lesion data from scans obtained 24–72 hours post-onset were delineated on high signal intensity (b = 1000) and transferred to a research version of RAPID automated software (Stanford University and iSchemaView) for post-processing. Apparent diffusion coefficient (ADC) maps and final ischaemic core volumes were automatically generated by the software.

**Acute Ischemic Stroke Workflow incorporating CT Perfusion regardless of NIHSS**
